# Editor's choice: COVID-19 and other infections in Africa

**DOI:** 10.4314/ahs.v23i1.1

**Published:** 2023-03

**Authors:** James K Tumwine

Welcome to this March 2023 issue of African Health Sciences, in which infectious diseases and sexual reproductive and child health issues take center stage. Rightly so, because infections are again in the news in this part of Africa: an outbreak of poliomyelitis in Burundi, and a simmering Marburg threat at the Tanzania Uganda boarder. South to South cooperation and collaboration is critical, as countries in the East African region have acquired hands-on knowledge and skills for handling epidemics of very infectious diseases. The experience of Uganda in this regard, is of special mention.

Be that as it may, we bring you papers on COVID-19^1^–^13^, followed by HIV^14^–^20^, hepatitis^21^–^22^, tuberculosis^23^–^24^, malaria^25^–^27^, and urinary tract infection^28^. Look out, particularly, for the paper on lessons learnt from the COVID-19 pandemic.

Sexual reproductive health issues also feature prominently in this issue. They range from intimate partner violence^29^–^30^, risky sexual behavior^31^; pathophysiology and management of pre-eclampsia^32^–^33^, and others. These include sexual health issues, pregnancy and its and outcome, gynecological conditions, and mental well-being^34^–^42^.

The non-communicable disease section includes papers on cancer^43^–^52^; cardiovascular disease^53^–^55^; and mental health^56^–^57^. The rest of the papers touch on several aspects of other non-communicable disease, surgery, training and health systems issues.

It only remains for me to thank all those who, in one way or another, contribute to AHS. We are very grateful to all our partners through the African Journal Partnership Program (AJPP), without whom our volunteer service as a totally Open Access journal, would not have been possible. Thank you for being our true and steadfast friends, as we strive to push publication of high-quality scientific papers in Africa to a higher level.
